# Mapping intratumoral heterogeneity in adolescent CNS tumors using simultaneous amide proton transfer-weighted signal and ^18^F-choline PET: A feasibility study

**DOI:** 10.1093/noajnl/vdag238

**Published:** 2026-09-09

**Authors:** Trung Nguyen, Marilena Rega, Francisco Torrealdea, Joe Hearle, Ananth Shankar, Xavier Golay, Harpreet Hyare

**Affiliations:** Children and Young People’s Cancer Services, University College London Hospitals NHS Trust, London, UK; Institute of Nuclear Medicine, University College London, London, UK; Centre for Medical Imaging, University College London, London, UK; University College London Medical School, London, UK; Children and Young People’s Cancer Services, University College London Hospitals NHS Trust, London, UK; Gold Standard Phantoms, Sheffield, UK; Department of Translational Neuroscience and Stroke, UCL Queen Square Institute of Neurology, London, UK; Department of Translational Neuroscience and Stroke, UCL Queen Square Institute of Neurology, London, UK

**Keywords:** APT-CEST, AYA, choline PET, glioma

## Abstract

**Background:**

Pediatric and adolescent gliomas and glioneuronal tumors remain challenging to assess noninvasively. Amide proton transfer (APT) chemical exchange saturation transfer (CEST) MRI has shown promise in adult gliomas but has not been well studied in younger patients. We conducted a feasibility study to assess whether APT-weighted signal can act as a noninvasive surrogate of tumor proliferation in adolescent central nervous system (CNS) tumors by correlating it with ^18^F-choline PET uptake (standardized uptake value [SUV]) as a proxy for membrane synthesis/proliferative activity.

**Methods:**

Ten adolescent patients (14-19 years), seven with histologically confirmed and three with radiologically suspected CNS tumors, underwent simultaneous APT-CEST and ^18^F-choline PET-MRI. Regions of interest (ROIs) corresponding to nonenhancing, enhancing, necrotic tumor, and contralateral white matter were delineated. Mean APT-weighted signal intensity and PET SUV were extracted per ROI. Nonparametric statistics and Spearman’s correlation analyses were performed.

**Results:**

APT-weighted signal was significantly elevated in enhancing, nonenhancing, and necrotic tumor ROIs compared to normal white matter (*P* < .001). ^18^F-choline SUV was elevated in enhancing and necrotic ROIs vs white matter, but not significantly so for nonenhancing tumor (*P* = .02). A correlation between enhancing and whole-tumor APT-weighted signal and ^18^F-choline SUV was seen (*P* < .001).

**Conclusion:**

This pilot feasibility study demonstrates that APT-CEST MRI is technically feasible in adolescents with CNS tumors and that APT-weighted signal may reflect tumor metabolic activity. The findings are hypothesis-generating and require validation in larger, histologically homogeneous cohorts. The detection of elevated APT-weighted signal even in nonenhancing tumor regions suggests potential utility in monitoring noncontrast-enhancing disease. Larger cohorts and multimodal correlation (eg Ki-67, amino acid PET) are warranted to confirm and extend these findings.

Key Points

- Amide proton transfer (APT)-weighted chemical exchange saturation transfer (CEST) MRI is technically feasible in adolescents with central nervous system tumors and can map intratumoral heterogeneity across enhancing, nonenhancing, and necrotic compartments.
- A correlation between enhancing tumor APT-weighted signal and ^18^F-choline PET standardized uptake value supports APT-weighted CEST MRI as a potential surrogate biomarker of tumor metabolic activity in this population.
- Elevated APT-weighted signal in nonenhancing tumor regions suggests sensitivity to infiltrative, noncontrast-enhancing disease that is not reliably captured by choline PET.

Importance of the Study

This is the first pilot study to pair simultaneous amide proton transfer (APT)-weighted chemical exchange saturation transfer (CEST) MRI and ^18^F-choline PET in adolescents with central nervous system (CNS) tumors, addressing a critical gap in pediatric neuro-oncology imaging. Unlike adult studies, the adolescent CNS tumor spectrum is particularly heterogeneous and noninvasive characterization of tumor biology remains limited. By demonstrating both technical feasibility and a strong imaging-metabolic correlation at the whole-tumor level, this work establishes APT-weighted signal as a candidate surrogate biomarker for proliferative activity in this age group. The detection of elevated APT-weighted signal in nonenhancing tumor regions, not captured by choline PET, positions APT-CEST as a potential complement to standard MRI for assessing infiltrative disease burden. Future prospective studies integrating histopathological validation, amino acid PET, and longitudinal follow-up are needed to translate these findings into clinical practice.

Central nervous system (CNS) tumors are the leading cause of cancer death in children, and gliomas remain a major challenge in the adolescent population.^1^^,^^2^ Imaging-based assessment of treatment response is impeded by pseudoprogression and pseudoresponse phenomena, limiting the reliability of conventional MRI (contrast enhancement, size) in many therapeutic contexts.^3^ Thus, there is a pressing need for advanced imaging biomarkers that go beyond morphology to probe tumor biology.

Chemical exchange saturation transfer (CEST) MRI techniques allow indirect imaging of exchangeable protons (eg amide protons) associated with endogenous mobile proteins and peptides, at higher spatial resolution than conventional spectroscopy.^4^ APT-CEST exploits the exchange between amide protons (resonating ∼3.5 ppm) and bulk water protons. Because malignant tissues tend to harbor elevated concentrations of mobile proteins and peptides—and potentially altered pH/microenvironment—APT-weighted signal may serve as a marker of tumor aggressiveness.^5-7^ In adults, APT-weighted signal has been correlated with histologic grade, molecular markers (eg IDH mutation/MGMT methylation), and survival outcomes^8-10^ in addition to differentiating tumor progression from pseudoprogression.^11^^,^^12^ However, whilst there are some studies showing value in differentiating pediatric tumor subtypes,^13^ the general adoption in pediatric and adolescent tumor imaging is sparse.

We hypothesized that APT-weighted signal might serve as a noninvasive biomarker of proliferation in adolescent CNS tumors, and that correlation with ^18^F-choline PET uptake (a marker of membrane turnover and proliferation) would provide preliminary validation.^14-16^ Although amino acid PET (eg ^11^C-MET, ^18^F-FET) is increasingly the clinical standard in glioma imaging,^3^^,^^17^^,^^18^ 18F-choline provides favorable spatial resolution and contrast for this proof-of-concept study.^19^

Recent reviews of CEST in gliomas emphasize that APT-weighted signal is sensitive not only to protein concentration but also to pH, magnetization transfer (MT) background, and saturation settings. Multipool modeling (eg Lorentzian fitting/REX metrics) may help disentangle competing contributions.^20^^,^^21^ A recent review highlights that higher APT-weighted signals tend to correlate with higher-grade gliomas, and that molecular phenotypes (eg IDH status) may modulate these signals in adult cohorts.^22^

Here, we present a feasibility study of concurrent APT-CEST and ^18^F-choline PET imaging in adolescents with CNS tumors, exploring the relationship between APT-weighted signal and PET uptake, and the ability of APT-weighted signal to characterize enhancing, nonenhancing, and necrotic tumor compartments, with whole tumor as a secondary analysis.

## Methods

### Patients

Ten adolescent patients (male *n* = 6, mean age 16.4 years, age range 14-19 years) were prospectively recruited for ^18^F-choline PET and APT-CEST between April 2016 and September 2019. Of these, 7 had histologically confirmed CNS tumors (on prior or subsequent biopsy/resection) and 3 were diagnosed radiologically as suspected gliomas based on conventional MRI characteristics at the time of imaging. Patient characteristics including histopathology and molecular genetics, treatment received, and clinical outcomes are summarized in Table 1. In all instances, APT-CEST and ^18^F-choline PET were performed simultaneously, thereby ensuring a good registration of images and accurate quantification as both scans were performed at the same time point.

**Table 1.** Main characteristics of patients

Patient	Sex	Clinical presentation	Histopathology	WHO grade	Molecular genetics	Treatment	Patient outcomes
1	F	Headache, vomiting, visual deterioration	GBM	IV	*IDH* mutant *ATRX* loss of expression *H3* negative	Near total resection Radiotherapy Temozolomide	Alive, well and in continuous remission
2	F	Seizures	Diffuse astrocytoma	II	*IDH* mutant *ATRX* loss of expression	Radiotherapy Temozolomide	Alive, well and in continuous remission
3	F	Visual deterioration	Midline HGG	IV	*IDH* wildtype *H3* negative	Radiotherapy Temozolomide	Alive, well and in continuous remission
4	M	Seizures	Pilocytic astrocytoma	I	*IDH* wildtype *ATRX* retained *BRAF* mutation	Complete resection	Alive, well and stable disease
5	M	Papilloedema, visual deterioration, ataxia	Midline HGG brainstem	IV	*H3* mutant *ATRX* retained	Radiotherapy Temozolomide PCV maintenance chemotherapy	Died of disease progression 15 months after diagnosis
6	M	Seizures	GBM	IV	Unknown	Reirradiation	Died of disease progression 10 months after recurrence
7	M	Headache, weakness	Diffuse midline glioma	IV	*IDH* wildtype *H3* mutant *ATRX* loss of expression *BRAF* negative	Radiotherapy Temozolomide	Alive with disease, terminally ill due to disease progression
8	F	Headache, seizures	Glioneuronal tumor	I	*IDH* wildtype *ATRX* retained	Debulking with residual disease, decompression of ventricle	Alive, well and in continuous remission
9	M	Headache, papilloedema	Schwannoma	I	*IDH* wildtype	None required	Alive, well and in continuous remission
10	M	Headache, coordination difficulties, ataxia	Rosette-forming glioneuronal tumor	I	*BRAF* negative	Temozolomide and lomustine	Alive, well and stable disease

Abbreviations: GBM, glioblastoma multiforme; HGG, high-grade glioma; PCV, procarbazine, lomustine, vincristine.

### Imaging Protocol

All PET studies were conducted on a Siemens mMR biograph with an 8-channel dedicated PET-MRI receive head coil. PET images were acquired 10 min following intravenous injection of ^18^F-choline with dose administered dependent on patient weight (3.5 MBq/kg up to a maximum of 250 MBq). Data were acquired for 30 min postinjection (matrix size 344 × 344) and corrected for scatter (relative scatter correction) and a 2-mm full width at half maximum Gaussian filter was applied. Attenuation correction was performed in 3 dimensions (3 iterations, 21 subsets) using the standard μ-map generated by the ultrashort echo time sequence on the PET-MRI. Standardized uptake value (SUV) images were calculated accounting for the time between injection and acquisition and the ^18^F-choline half-life.

PET data were acquired simultaneously with the MRI sequences under standard resting conditions. The MRI protocol included an axial T_2_-weighted (w) inversion-recovery acquisition (TE/TR 397/5000, slice thickness (st) 4 mm), an axial T_2_w turbo-spin-echo sequence (TE/TR 300/4000, st 4 mm), and an axial T_1_w 3D isovolumetric sequence before and after gadolinium (0.2 ml/kg) injection (TE/TR 3.8/2000, voxel size 1.1 × 1.1 × 1.1 mm^3^). Diffusion-weighted imaging (TE/TR 100/8000, st 5 mm) was acquired using a single-shot echo-planar sequence in the axial plane with 3 *b*-values (0, 500, and 1000 s/mm^2^).

Chemical exchange saturation transfer was acquired at 2 *B*_1_ saturation powers (0.75 and 1.25 μT equivalent power) to allow for *B*_1_ field homogeneity correction with a snapshot CEST acquisition. Saturation was achieved with a 3 s pulse train (Gaussian shaped pulses, 50 ms duration each, 50% duty cycle). A full Z-spectrum was acquired for each *B*_1_ power with 41 frequency offsets ranging between ±5 ppm, sampled in alternative mode from −5 ppm to 0 ppm. A reference scan was also acquired at the start of the sequence for normalization purposes. Saturation was followed by a single-shot centric-spiral reordered 3D gradient echo readout with an 8º flip angle, TE = 1.57 ms, TR = 1.57 ms, GRAPPA factor 2 in the phase encode direction and a matrix size 108 × 112 × 8. The effective TR per frequency offset was 5.48 s (accounting for both the readout and the saturation times).

A WASAB1 scan was acquired and was used for field homogeneity corrections. The WASAB1 preparation consisted of 1 rectangular pulse of 5 ms duration and *B*_1_ = 3.7 μT. A reference scan was acquired for normalization purposes and for 31 frequency offsets ranging from −3 to 3 ppm in alternative mode. The readout used was the same as for the CEST acquisition and was followed by a 2-s delay (TR per frequency was 4.49 s).

Z-spectra were corrected for *B*_0_ and *B*_1_ inhomogeneities and normalized at the reference. Amide proton transfer-weighted images were calculated as the Z-spectra asymmetry centered at 3.5 ppm (from 3.2 to 3.8 ppm), also known as the APT range.

### Image Analysis

#### Qualitative

An experienced neuroradiologist (H.H.) assessed the conventional MRI scans using 5 criteria from the Visually AcceSAble Rembrandt Images (VASARI) MRI feature set schema (http://wiki.nci.nih.gov/display/CIP/VASARI): (1) tumor location, (2) size of tumor epicenter, (3) enhancement quality, (4) proportion enhancing of whole tumor, and (5) proportion necrosis. The maximal perpendicular diameters (mm) were also recorded. The ^18^F-choline SUV and APT-weighted images were scored independently as positive if there was focal uptake within the tumor that was greater than normal brain activity.

#### Quantitative

Region of interest delineation was performed by an experienced neuroradiologist (H.H.) using ITKsnap (version 3.8; http://www.itksnap.org). T_2_-FLAIR and T_1_w-post-Gd images were used to draw ROIs of the whole tumor, including “nonenhancing,” “enhancing,” and “necrotic core” in the tumor, and healthy contralateral “white matter.” Areas with obvious artifact or signal from blood vessels were excluded from the ROI. The APT-weighted and ¹⁸F-choline SUV maps were coregistered to the T_1_w postcontrast reference image using rigid-body affine registration (6 degrees of freedom); registration accuracy was visually verified for each case. Mean APT-weighted signal intensity (APT_mean_) and mean SUV (SUV_mean_) per ROI were extracted using ITKsnap’s ROI statistics function.

### Statistical Analysis

All values are expressed as mean (standard deviation). The APT_mean_ and SUV_mean_ in the normal appearing white matter, enhancing, nonenhancing and necrotic core ROIs were compared using the Kruskal-Wallis test. Post hoc tests were performed comparing each of the enhancing, nonenhancing and necrotic core ROIs to the corresponding normal appearing white matter ROI. Due to the number of calculations, the significance level was set at *P* < .01. Correlations between APT_mean_ and SUV_mean_ in the whole tumor, enhancing, nonenhancing, and necrotic core were assessed using the Spearman’s correlation coefficient. Statistical analyses were performed using SPSS Statistics (IBM Version 19.0). Because multiple ROI types were obtained per patient, ROI-level observations within the same patient were not treated as fully independent; correlations used patient-level means as the primary unit of analysis. The prespecified significance threshold of *P* < .01 for post hoc pairwise comparisons accounts for multiplicity of testing. The small sample size limits statistical power and effect size estimates may be inflated; all findings should be regarded as exploratory and hypothesis-generating.

## Results

### Qualitative Findings

#### Conventional MRI

The appearances of the tumors on conventional MRI were variable. Most tumors (*n* = 7) were supratentorial and unilateral in location, with 3 posterior fossa tumors that were central. Most tumors (*n* = 8) demonstrated some degree of enhancement, with 6 demonstrating marked/avid enhancement and 2 demonstrating mild enhancement. Seven tumors demonstrated necrosis ranging from <5% to 33% necrosis, and all but 2 tumors demonstrated a nonenhancing component. The conventional imaging features for all patients are summarized in Table 2 according to the VASARI MRI feature set.

**Table 2.** Conventional MRI, 18F-choline SUV, and APT-weighted SI findings

Patient	^18^F-Cho PET SUV	APT-weighted SI	VASARI MRI features						
			F1 location	F2 side	F4 enhancement	F5 % enhancement	F7 % necrosis	Longest axis (mm)	Shortest axis (mm)
1	+	+	Temporal	R	Marked/avid	67-95	6-33	32.8	14.8
2	−	+	Frontal	R	None	0	0	33.1	21.9
3	−	+	Pons	C	None	0	0	28.8	28.4
4	+	+	Temporal	L	Marked/avid	6-33	<5	58.8	35.7
5	+	+	Pons	C	Minimal/mild	6-33	6-33	49.0	34.8
6	+	+	Frontal	L	Marked/avid	6-33	6-33	142.9	69.4
7	+	+	Thalamus	L	Minimal/mild	<5	0	11.9	8.4
8	+	+	Parietal	L	Marked/avid	34-66	<5	27.1	21.2
9	+	+	Frontal	R	Marked/avid	>95	<5	50.1	30.4
10	+	+	Cerebellum	C	Marked/avid	>95	<5	35.8	20.6

Abbreviations: C, central; L, left; R, right; SI, signal intensity; +, increased signal in the tumor compared to normal brain; −, no increased signal in the tumor compared to normal brain.

#### ^18^F-Choline PET-MRI and APT-Weighted Signal

Most tumors (*n* = 8) demonstrated ^18^F-choline PET avidity while all tumors demonstrated increased signal within the tumor compared to normal brain on the APT-weighted image. The 2 tumors that were ^18^F-choline PET SUV negative were also nonenhancing tumors. Examples of the SUV and APT-weighted maps obtained in 3 of the patients are demonstrated in Figure 1.

**Figure 1.**
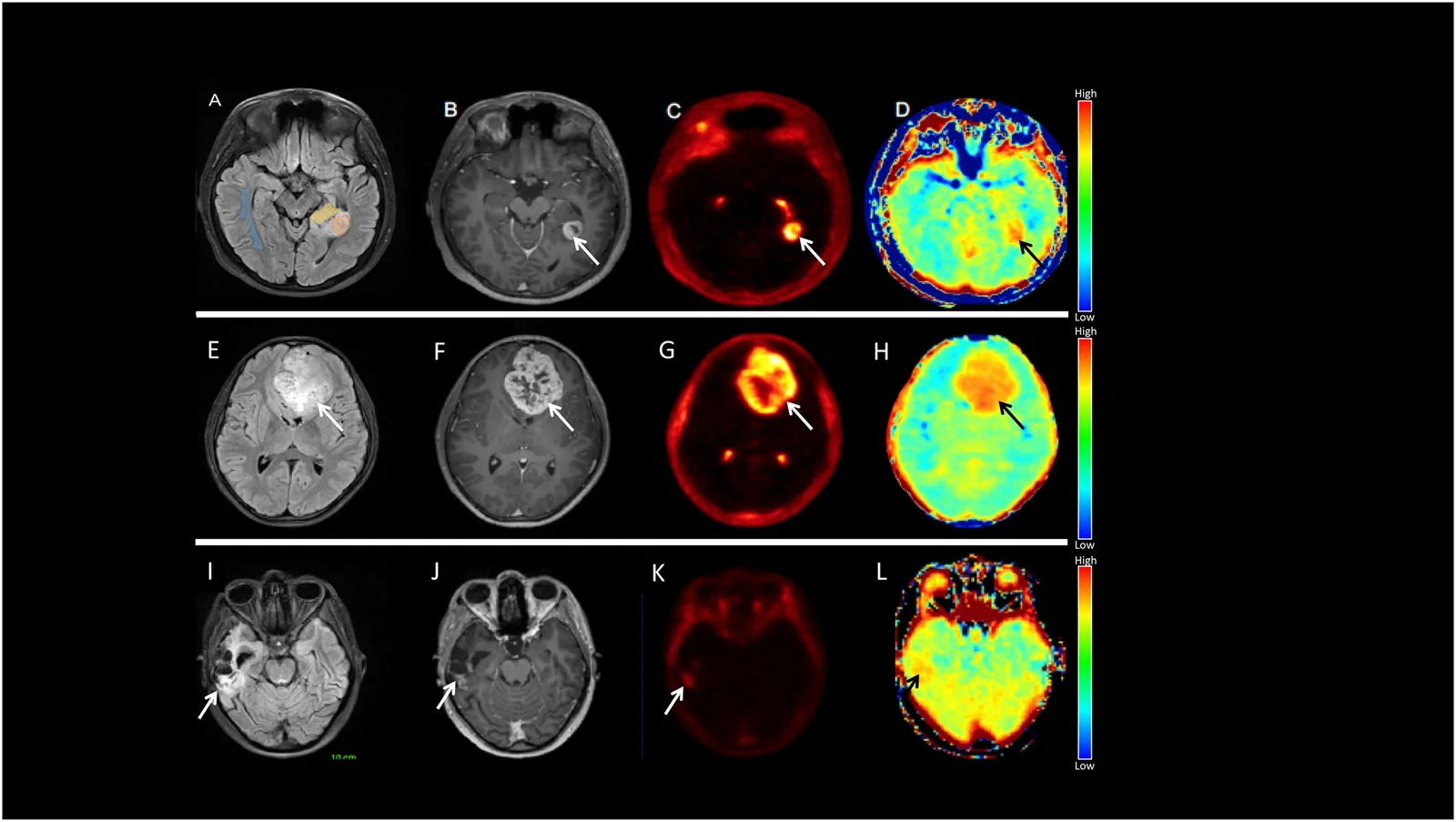
Examples of conventional MRI, ^18^F-choline SUV, and APT-weighted maps in 3 of the patients. Case 2 (A-D) represents a 14-year-old male with a pilocytic astrocytoma (WHO I) (A) FLAIR image demonstrates the ROIs: red = enhancing, orange = nonenhancing, blue = normal appearing white matter. (B) T_1_w image postcontrast demonstrates ring enhancing lesion, (C) increased 18F-choline SUV, and (D) increased APT-weighted signal in the enhancing component. Case 5 (E-H) represents a 16-year-old male with a schwannoma (WHO I). FLAIR image demonstrates a large midline lesion associated with avid heterogenous enhancement, increased ^18^F-choline SUV, and increased APT-weighted signal throughout the tumor. Case 6 (I-L) represents a 19-year-old female postresection of a GBM (WHO IV). FLAIR image demonstrates the right temporal surgical bed with increased signal abnormality at the posterior margin associated with mild nodular enhancement, focal increased ^18^F-choline SUV, and focal increased APT-weighted signal within the enhancing component.

### Quantitative Findings

#### Tumor ROI Analysis

The overall means and standard deviations of the APT-weighted signal and ^18^F-choline PET SUV in each ROI for the patients are summarized in Table 3. The highest APT-weighted signal was seen in the enhancing and necrotic core ROI and the lowest APT-weighted signal was seen in the nonenhancing tumor ROI (Figure 2). The highest ^18^F-choline PET SUV was observed for the enhancing tumor ROI and the lowest was seen in the nonenhancing tumor ROI. There was a significant difference in APT-weighted signal for the enhancing, nonenhancing and necrotic ROI when compared to normal appearing white matter (*P* < .001). Although, there was a significant difference in ^18^F-choline PET SUV for the enhancing and necrotic ROI compared to normal appearing white matter (*P* < .001), there was no significant difference in ^18^F-choline PET SUV for the nonenhancing ROI vs normal appearing white matter (Figure 2, *P* = .02).

**Figure 2.**
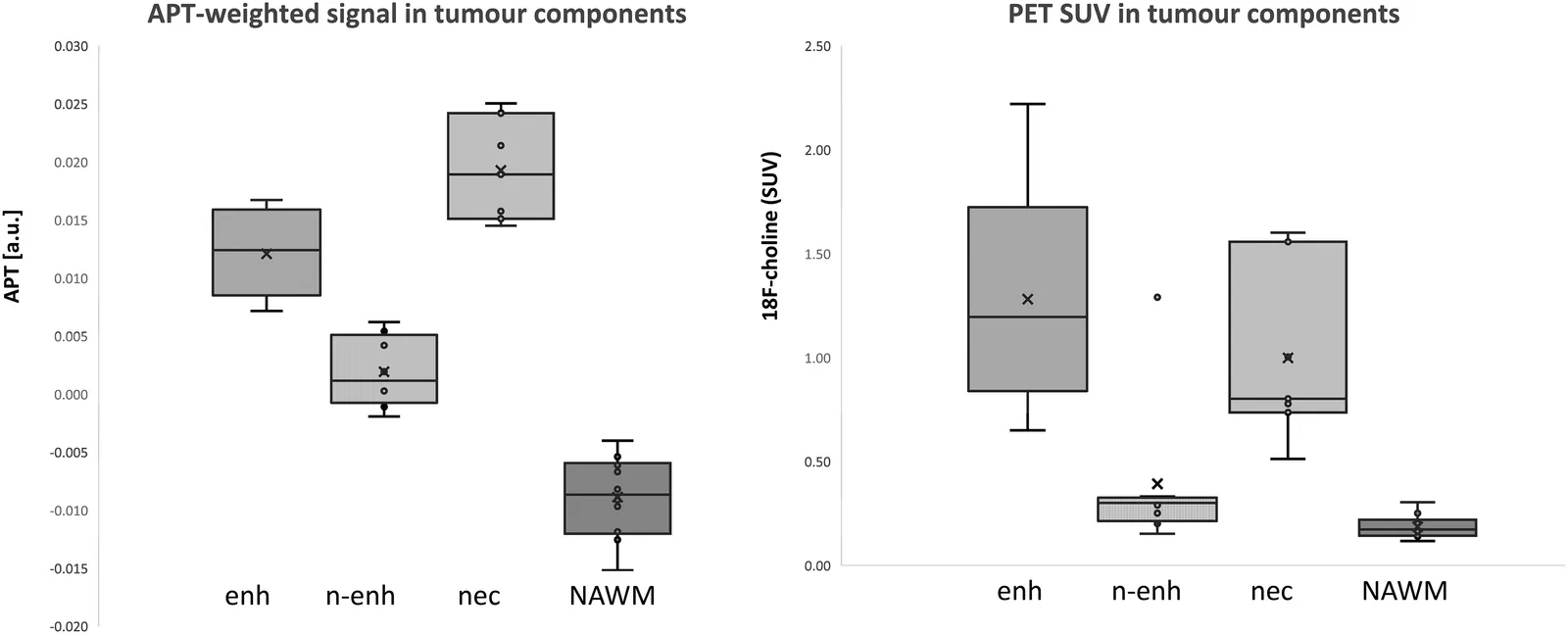
APT-weighted signal and 18F-choline PET SUV in enhancing, nonenhancing, necrotic and healthy appearing white matter ROI. Box plot demonstrating APT-weighted signal and ^18^F-choline PET SUV in the enhancing, nonenhancing, necrotic core and normal appearing white matter ROI. Abbreviations: APT, amide proton transfer; a.u., arbitrary units; enh, enhancing ROI; ^18^F-choline, fluoromethylcholine; NAWM, normal appearing white matter ROI; n-enh, nonenhancing ROI; nec, necrotic core ROI; SUV, standardized uptake value.

**Table 3.** Summary of quantitative findings in tumor ROI

ROI	APT (a.u.)	^18^F-cho PET (SUV)
Enhancing	0.012 (0.004)	1.281 (0.528)
Nonenhancing	0.002 (0.003)	0.392 (0.368)
Necrotic core	0.019 (0.004)	0.998 (0.422)
Normal appearing white matter	−0.009 (0.004)	0.186 (0.057)
*P*-value enhancing vs normal appearing white matter	<.001	<.001
*P*-value non-enhancing vs normal appearing white matter	<.001	.02
*P*-value necrotic core vs normal appearing white matter	<.001	<.001

All values are expressed as mean (standard deviation).

Abbreviations: APT, amide proton transfer; a.u., arbitrary units; ^18^F-cho, ^18^F-fluoromethylcholine; ROI, region of interest; SUV, standardized uptake value.

#### Correlation of APT-Weighted Signal and ^18^F-Choline PET SUV

The correlation between the different metrics was also assessed using the Spearman’s correlation coefficient. A correlation was observed for enhancing tumor ROI APT-weighted signal vs ^18^F-choline PET SUV (Spearman *ρ * = 0.63, *P* < .001) but no correlations were found between nonenhancing or necrotic ROI APT-weighted signal and ^18^F-choline PET SUV. Whole-tumor ROI APT-weighted signal when compared to whole-tumor ^18^F-choline PET SUV demonstrated a correlation (Spearman *ρ * = 0.86, *P* < .001). Figure 3 demonstrates the relationship between APT-weighted signal and ^18^F-choline PET SUV in the enhancing and whole-tumor ROIs.

**Figure 3.**
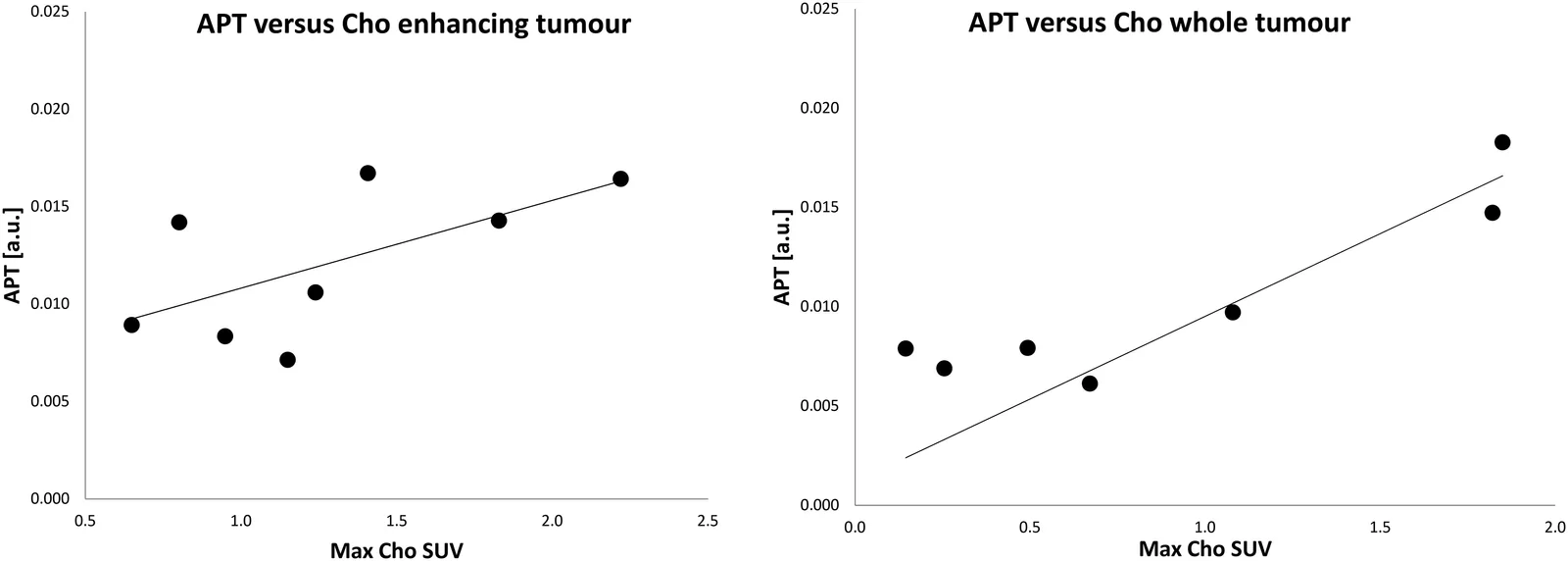
Relationship between APT-weighted signal and ^18^F-choline SUV in the enhancing and whole-tumor ROIs. Relationship between APT-weighted signal and ^18^F-choline PET SUV in the enhancing tumor and whole-tumor ROIs: enhancing tumor ROI (Spearman *ρ* = 0.63, *P* < .001), whole-tumor ^18^F-choline PET SUV (Spearman *ρ* = 0.86, *P* < .001). Abbreviations: APT, amide proton transfer; a.u., arbitrary units; Cho, fluoromethylcholine; enh, enhancing ROI; NAWM, normal appearing white matter ROI; n-enh, nonenhancing ROI; nec, necrotic core ROI; SUV, standardized uptake value.

## Discussion

We demonstrate the technical feasibility of APT-CEST in pediatric and adolescent CNS tumors and demonstrate that APT-weighted signal can map intratumoral heterogeneity. We demonstrate a correlation with ^18^F-choline PET uptake at the enhancing and whole-tumor level, suggesting that APT-weighted signal is a sensitive biomarker of tumor proliferation. It must be emphasized that these findings derive from a small, histologically heterogeneous pilot cohort; effect size estimates may be inflated and the results should be regarded as hypothesis-generating pending validation in larger, tumor-type-specific series. Importantly, elevated APT-weighted signal was demonstrated in nonenhancing tumor regions suggesting potential sensitivity to noncontrast-enhancing infiltrative disease.

In adult glioma cohorts, APT-CEST has been explored extensively. Chemical exchange saturation transfer was first used in rat models to differentiate normal brain tissue from glioma tissue,^23^ predict histological grading of gliomas and to assess therapeutic response to temozolomide in murine models.^24^ Studies in human adults also suggest CEST might be useful for predicting histological grade of gliomas,^6^^,^^7^,^25^^,^^26^ IDH mutation status/MGMT promoter methylation status,^9^^,^^26^ assessing response to treatment^10^^,^^27^ and be an independent predictor of poor overall survival and progression-free survival in high-grade glioma.^28^

A major challenge for clinical adoption is reproducibility. A recent multiscanner/multisession reproducibility study (19 healthy volunteers, 7 brain tumor patients) at 3T reported low coefficients of variation (COVs) for Lorentzian difference (LD) and relaxation-compensated metrics (MTR_Rex) (within-session WM COV ∼0.56%, between sessions ∼0.84%) and somewhat higher variability in tumor ROIs (∼4%-6%) but acceptable stability across sessions and scanners.^29^ This suggests that advanced fitting methods (beyond simple MTR_asym) may offer more robust quantification, particularly for longitudinal tracking. Another study^21^ evaluated semisolid MT and APT-based metrics (asymmetry, Lorentzian fits) to predict clinical endpoints and argued that combining CEST and MT modeling helps isolate biologically meaningful contrast while suppressing confounds (MT, spillover). It is important to note that the APT-weighted signal reported here was derived using MTR asymmetry at 3.5 ppm with saturation powers of 0.75 and 1.25 μT. At these *B*_1_ levels, the asymmetry metric reflects a composite CEST contrast in the APT frequency range rather than a pure measure of amide proton transfer: contributions from nuclear Overhauser effects (NOE), semisolid MT background, and direct water saturation are expected to remain substantial. Current consensus^30^ recommends either higher saturation power (∼2 μT) with MTR_asym, or model-based approaches such as LD analysis or relaxation-compensated metrics (MTR_Rex) for improved amide specificity. The pragmatic rationale for the simpler asymmetry approach here is that it was the clinically validated pipeline at our institution during the acquisition period (2016-2019), and the raw Z-spectrum data are no longer available for retrospective reprocessing. All interpretations of APT-weighted signal in the present manuscript should therefore be understood in this context, and we recommend that prospective follow-up studies adopt model-based quantification from the outset.

Recently, APT-CEST has also been applied to the problem of distinguishing glioma recurrence from pseudoprogression. A 2025 study in recurrent glioma patients (30 progression, 12 pseudoprogression) showed that APT-weighted signal was significantly higher in progressing disease than in pseudoprogression (1.99% vs 1.26%, *P* = .008). Additionally, APT-weighted signal revealed focal “metabolic hotspots” within FLAIR lesions not evident on conventional MRI, and early changes in resection cavity APT-weighted signal even before contrast changes.^11^ This supports the potential of APT-CEST for treatment monitoring beyond static imaging.

In pediatric neuro-oncology, APT-CEST remains nascent. A brief initial experience paper outlines technical challenges (motion, *B*_0_/*B*_1_ inhomogeneity, smaller brain size) and potential value, but few case series exist. Meanwhile, reviews of advanced neuroimaging in pediatric brain tumors list CEST and molecular MRI among the promising emerging modalities.^31^

To our knowledge, this is the first study to pair APT-weighted signal with ^18^F-choline PET uptake in adolescent CNS tumors. The strong correlation at the whole-tumor level supports the hypothesis that APT-weighted signal is sensitive to tumor proliferation/metabolic turnover. Increased ^11^C-choline (Cho) PET SUV has been shown to correlate with cell proliferation indices in lung cancer biopsy specimens, thought to be due to overexpression of choline kinase in cancer cell membranes,^15^^,^^32^^,^^33^ with good tumor to normal brain contrast and high spatial resolution. The limitations of ¹⁸F-choline relative to amino acid tracers should be acknowledged: choline PET has lower specificity for diffuse infiltrative low-grade disease, is susceptible to uptake in inflammatory tissue, and provides inferior tumor margin delineation compared to ¹⁸F-FET, ¹¹C-MET, and ¹⁸F-DOPA. Future studies pairing APT-weighted signal with amino acid PET would provide stronger biological linkage and direct relevance to current clinical glioma imaging practice.

The observation of elevated APT-weighted signal in necrotic ROIs is intriguing but importantly cannot be attributed to viable tumor proliferation. Multiple biophysical processes may contribute to this finding: (1) accumulation of mobile peptides and protein degradation products from cellular breakdown; (2) altered pH in the necrotic microenvironment; (3) increased free water content; (4) MT background effects not captured by simple MTR asymmetry; and (5) spillover artifact from direct water saturation, a recognized major confound in APT-CEST measurements in necrotic tissue.^34^ Given these confounds, the primary correlation analysis between APT-weighted signal and ¹⁸F-choline PET SUV is presented for the enhancing and nonenhancing tumor compartments only; the whole-tumor correlation is reported as a secondary, exploratory finding with appropriate caveats. Future studies should prospectively exclude necrotic cores from primary APT biomarker analyses or apply model-based correction for spillover.

The nonenhancing tumor ROI showed elevated APT-weighted signal vs white matter, in line with other studies^35^ whilst ¹⁸F-choline PET uptake did not differ significantly. This may stem from limited sensitivity of ¹⁸F-choline PET in diffuse infiltrative disease, partial volume effects, or mismatch between proliferation and choline metabolism in lower-density regions. Indeed, image-targeted biopsies to correlate with cell density would strengthen claims of APT-weighted signal as a proliferative biomarker. Alternatively, amino acid PET tracers, ^11^C-MET, ^18^F-FET, and ¹⁸F-DOPA specific for glioma metabolic activity,^36^ may provide improved correlation with CEST signals. While there are several studies comparing APT-CEST with methionine,^37^ one of which showed higher diagnostic performance for APT compared to methionine for posttreatment gliomas,^38^ future larger simultaneous APT-CEST/amino acid PET studies could provide more direct biological linkage.^17^

The potential clinical applications of APT-CEST in adolescent neuro-oncology, while requiring prospective validation, are worth noting. First, the detection of elevated APT-weighted signal in nonenhancing tumor regions that are not reliably captured by choline PET suggests a role in delineating infiltrative disease and guiding image-targeted biopsy. Second, APT-CEST may complement conventional MRI surveillance by providing a noncontrast-enhancing tumor burden metric. Third, treatment response monitoring represents a promising application: emerging data in adult glioma demonstrate that APT-weighted signal changes precede conventional imaging changes, and the ability to distinguish true progression from pseudoprogression^11^ is of direct clinical relevance. These applications are framed as exploratory and must be validated in prospective pediatric/adolescent cohorts before clinical translation.

In summary, this study demonstrates the feasibility and translational relevance of APT-CEST MRI in adolescents with CNS tumors and provides the first evidence of an association between APT-weighted signal and PET-derived metabolic activity in the enhancing tumor population. Beyond corroborating proliferative burden at the global tumor level, APT-CEST uniquely identified abnormal signal in nonenhancing tumor compartments that were not reliably captured by choline PET, highlighting its sensitivity to biologically active disease beyond conventional imaging contrast. These findings position APT-CEST as a promising adjunct to standard MRI for characterizing intratumoral heterogeneity and assessing noncontrast-enhancing tumor burden in adolescent neuro-oncology. Future prospective studies integrating histopathology, amino acid PET, and longitudinal imaging will be essential to validate its role as a robust biomarker for disease monitoring and treatment response.

## Conclusion

This pilot study presents the first simultaneous correlation of APT-weighted CEST signal and ^18^F-choline PET in adolescent CNS tumors, demonstrating feasibility, compartmental discrimination, and a strong imaging-metabolic correlation. We suggest that APT-CEST may serve as a surrogate biomarker of tumor proliferation and could aid in assessing nonenhancing disease activity. Further prospective studies integrating amino acid PET, histology, and longitudinal follow-up are warranted to validate and refine the role of APT-CEST in pediatric neuro-oncology.

## Data Availability

All data are available upon reasonable request to the corresponding author.
